# Biological Markers Predictive of Invasive Recurrence in DCIS

**Published:** 2008-01-22

**Authors:** Sharon Nofech-Mozes, Jacqueline Spayne, Eileen Rakovitch, Harriette J Kahn, Arun Seth, Jean-Phillippe Pignol, Lavina Lickley, Lawrence Paszat, Wedad Hanna

**Affiliations:** 1 Department of Pathology, Sunnybrook Health Sciences Center (SHSC); 2 Department of Radiation Oncology Toronto Sunnybrook Regional Cancer Centre; 3 Department of Molecular Biology, SHSC; 4 Research Institue, SHSC; 5 Department of Surgery, SHSC; 6 The Institute for Clinical Evaluative Sciences

**Keywords:** breast cancer, ductal carcinoma in situ, molecular markers, biological markers, HER2/neu, tumour invasion, prognosis, recurrence

## Abstract

DCIS is a heterogeneous group of non-invasive cancers of the breast characterized by various degrees of differentiation and unpredictable propensity for transformation into invasive carcinoma. We examined the expression and prognostic value of 9 biological markers with a potential role in tumor progression in 133 patients with pure DCIS treated with breast conserving surgery alone, between 1982–2000. Histology was reviewed and immunohistochemical staining was performed. Pearson correlation coefficient was used to determine the associations between markers and histopathological features. Univariate and multivariate analysis examined associations between time to recurrence and clinicopathologic features and biological markers.

Median age at diagnosis was 55 years (25–85). With a median follow up of 8.91 years, 41/133 patients recurred (21 as invasive recurrence). In this cohort 13.5% had low, 43% intermediate and 42% high nuclear grade. Comedo necrosis was found in 65% of cases. Expression of ER (62.4%), PR (55.6%), HER2/neu (31.6%), MIB1 (39.8%), p53 (22.6%), p21 (39.8%), Cyclin D1 (95.5%) calgranulin (20.5%), psoriasin (12%), was found in DCIS. HER2/neu was overexpressed in 45% that recurred as DCIS and 42.9% that recurred as invasive cancer, and only in 26.1% in cases that never recurred. On univariate analysis, HER2/neu overexpression was the only marker associated with an increased risk for any recurrence (p = 0.044). The hazard ratio for recurrence for HER2/neu positive DCIS was 1.927 (confidence interval 1.016–3.653) compared to HER2 negative DCIS. On multivariate analysis, HER2/neu overexpression remained the only independent variable significantly associated with any recurrence (p = 0.014) and with invasive recurrence (p = 0.044).

This data suggest that HER2/neu testing may become an important parameter in the management of DCIS and the treatment of cases with positive HER2/neu status could be modified accordingly, similar to the current approach for HER2/neu positive invasive disease.

## Introduction

With the adoption of screening mammography, the incidence of ductal carcinoma in situ (DCIS) has risen dramatically(1). DCIS is a heterogeneous group of pre-invasive cancers of the breast characterized by various degrees of differentiation and propensity for transformation into invasive carcinoma. Approximately 16–22% of DCIS treated with breast conserving surgery (BCS) alone will develop recurrence. Approximately half of them recur in the form of invasive disease which is associated with significantly reduced overall survival; whereas, the other half recur as DCIS(2). Postoperative radiotherapy reduces the recurrence rate to 7–9%(3–5), of which about half are invasive (4;6). In a large series with 15 years follow up, the first local failure rate was 16%, 2/3 as an invasive cancer, even with adjuvant radiotherapy (7). Currently our ability to identify this subset of patients that are at risk for developing invasive cancer is limited. Therefore, the standard of care for all patients diagnosed with DCIS is BCS followed by adjuvant radiation therapy. Clinical and histologic characteristics that may predict risk of local recurrence in women with DCIS have been identified in several studies (8–11). These include the presence of comedo necrosis, architectural subtype, tumor size and width of resection margins (11–21). However, the light microscopic examination in DCIS does not accurately identify patients at high risk for recurrence as invasive cancer. In recent years, there have been efforts to examine the value of different biological markers as prognostic and predictive factors in DCIS. Most of the molecular changes that characterize invasive breast cancer are already present in DCIS, though the lesion has not assumed a fully malignant phenotype (10;22–25). Previous studies found that the expression of the estrogen receptor (ER), progesterone receptor (PR), HER2/neu, Ki67 a proliferation marker, P53, and Bcl-2 correlate with tumor grade and are interrelated, but nevertheless do not add predictive or prognostic information (26–28). Experimental markers that contribute to various mechanisms leading to the complex pathway of tumor progression have been proposed. These include: 1) markers of tumor proliferation and cell cycle regulation (mitosin, telomerase, Cyclin D1, p21, IGFBP-rP1), 2) intercellular interaction (P cadherin, calgranulin, psoriasin), 3) extracellular matrix regulation (urokinase plasminogen activator system, metalloproteinase and its inhibitors) and 4) angiogenesis.

We identified the need for a study based on a large number of cases of pure DCIS who underwent uniform treatment and had long follow up. Based on our recent review of the literature (29), we conducted a study that focused on prognostic markers with an emphasis on invasive recurrence. A panel of markers with a potential role in tumor progression that could be evaluated by immunohistochemical methods was selected. These include estrogen and progesterone receptors, HER2/neu overexpression; proliferation marker: MIB1; molecules involved in cell cycle regulation: cyclin D1, p21, p53; and two calcium binding proteins: calgranulin and psoriasin.

The aim of this study was to identify novel pathological predictors of invasive recurrence to enable targeting an effective treatment to those at risk. Unnecessary treatment for most women with DCIS who will never develop invasive disease could be minimized.

## Materials and Methods

### Cohort

The study population comprised of 254 patients referred for surgical management of pure DCIS to one cancer centre between 1982 and 2000. We excluded 100/254 patients that were managed by mastectomy and those who received adjuvant radiation, as these treatments are known to reduce the recurrence rate significantly. In 133/154 patients with BCS and negative margins, we reviewed the original pathology and selected blocks containing representative tumor.

Demographic, treatment and outcome details were extracted from patient charts. The endpoint in this study was the development of recurrence, whether it was invasive or in situ carcinoma, at least six months after the BCS. All ipsilateral in situ or invasive carcinoma of ductal origin that was diagnosed after the surgical treatment including re-excision or completion mastectomies for positive margins were completed. Any event prior to that was attributed to incomplete excision.

### Pathology review

A complete pathology review was undertaken by one pathologist (SNM). A second pathologist (WH) reviewed 20% of the cases to assure at least 90% agreement as per the predefined protocol. In cases with grossly identified mass, the entire lesion was submitted, while in cases with no gross disease, the sectioned specimen was radiographed and areas of calcification or architectural distortion were sampled. The mean number of blocks per case was 15. Nuclear grade was determined using the Holland classification(30). In lesions with mixed patterns, we classified the lesion based on the higher grade. Histological subtype, presence of comedo necrosis, presence of invasion, the margin’s status and size were also documented. Comedo necrosis was considered present for any architectural pattern of DCIS in which a central zone of necrotic debris with karyorrhexis was identified. Tumors were divided into 2 groups by the presence or absence of any amount of comedo necrosis. Margins were called positive when DCIS was present on the inked or cauterized edge of the specimen. As per our cohort definition, cases with positive margins were excluded. As a result of the retrospective nature of this study, the size of the lesion could be accurately defined only in 81 specimens.

### Immunohistochemistry (IHC)

A panel of 9 antibodies against: estrogen receptors, progesterone receptors, HER2/neu oncoprotein, MIB1, cyclin D1, p21, p53, calgranulin, psoriasin; was used for immunohistochemistry on formalin fixed paraffin embedded tissue sections, following the manufacturer’s instructions (Table 1). A positive and negative control for each antibody was included in every run. The positive control for psoriasin and calgranulin consisted of 2 cases of DCIS with an invasive component that were included in a previous study. In these cases, positive psoriasin and calgranulin immunostains correlated with their gene upregulation as shown using DNA microarray technique(31). Nuclear staining was scored for ER, PR, MIB1, p53, p21, cyclin D1 and membranous staining for HER2/neu oncoprotein, and both nuclear and cytoplasmic staining were scored for calgranulin and psoriasin. HER2/neu immunostain was scored from 0 to 3+ as per the HerceptTest^TM^ scoring method. In equivocal cases, HER2/neu gene amplification was determined by chromogenic in situ hybridization (CISH). This was performed using the Zymed SPoT-Light^®^ HER2 CISH^TM^ Polymer Detection kit (84–0146). HER2/neu gene amplification was determined when there were six or more signals per nucleus or when clusters were identified in the tumor cells’ nuclei. The results of all the other immunohistochemical markers were recorded as continuous variables based on the proportion of positive tumor cells (0%–100%) regardless of their staining intensity. All the involved ducts on the slides were scored. For ER and PR a 10% cut-off value for positivity was used to categorize cases into positive or negative.

We compared the morphology and the expression of ER, PR, HER2/neu oncoprotein in the primary DCIS and subsequent recurrent tumor in the same patients, whether those were in situ or invasive recurrences. In 36/41 cases, blocks with representative recurrent tumors were available, these were stained for ER, PR and HER2/neu.

One pathologist (SNM) evaluated all immunostains results. Another pathologist (WH) reviewed 20% of the stains, including all equivocal HER2/neu, and the agreement level was greater than 90%. Both the retrospective pathology case review and the IHC evaluation were undertaken by the evaluator who was unaware of the results of the other IHC stains and of the patient’s outcome.

### Statistical Methods

The data was collected in an Access database. For comparison of proportions between the groups, the chi-square test was used and differences between 2 means were assessed with Student t-test for unpaired data. Statistically significant differences were assumed when p < 0.05. SPSS for Windows version 12 (SPSS Inc.) was used for statistical calculations. The Pearson correlation coefficient was used to determine associations between markers and histopathological features. Univariate survival analysis examined associations between time to recurrence such as in situ and/or invasive carcinoma and demographic (age) or pathologic (nuclear grade, comedo necrosis) features as well as expression of biological markers. Multivariate survival analysis with the Cox proportional hazards model was used to identify independent variables associated with recurrence.

Ethics approval for this study was obtained from the Research Ethics Board of the Toronto Academic Health Sciences Council.

## Results

The median age at diagnosis was 55 years (range 25–85). There was no significant correlation between age and outcome. The median follow up period was 8.9 years. During the follow up period 41/133 patients (30.8%) had a histologically documented recurrence, 20 (15%) cases as DCIS and 21 (15.8%) cases as invasive carcinoma. The median time to recurrence was 2.7 years, and 5.6 years for DCIS and invasive recurrence respectively.

In this cohort, 13.5% were classified as low grade, 43.6% intermediate and 42.9% high nuclear grade. Comedo necrosis was found in 65% of cases and unequivocal microinvasion was found in 3% of cases.

There were no significant associations found between recurrence rate (analyzed for any type of recurrence or specifically for invasive recurrence) and nuclear grade or comedo necrosis (Table 2).

**Figure 1:**
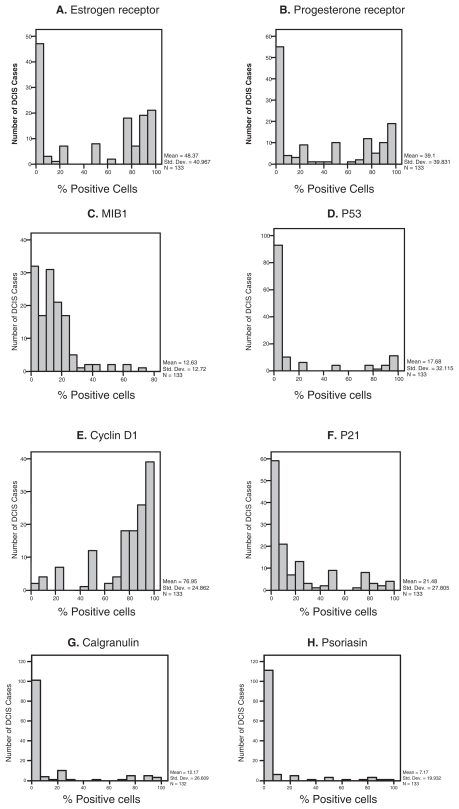
The distribution of positive DCIS cases according to the percentage of positive cells for each of the biological markers.

**Figure 2:**
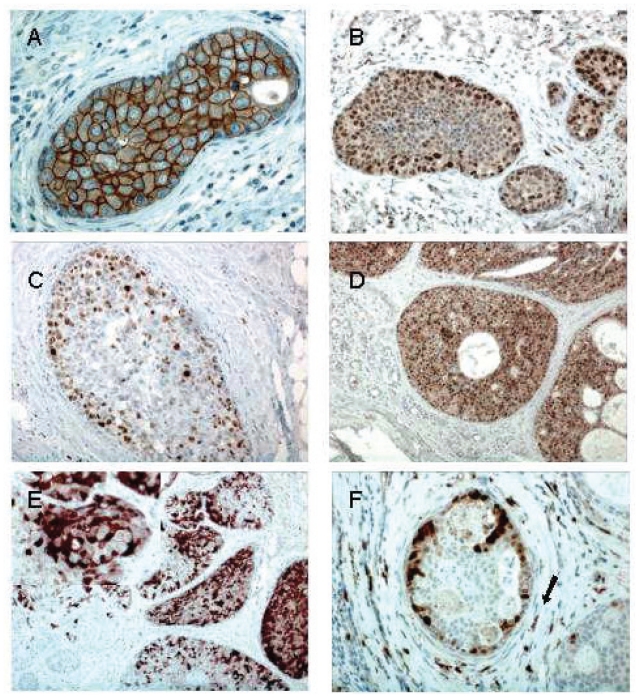
**2A**- Positive membranous immunostain for HER2/neu oncoprotein in high grade DCIS (clone: CB11, X200) **2B**- Positive nuclear immunostain for p21in 50% of the cells in intermediate grade DCIS. Note the variable intensity of stains. (x100) **2C**- Positive nuclear immunostain for MIB1 in 30% of the cells in high grade DCIS. (x100) **2D**- Positive nuclear immunostain for cyclin D1 in 100% of the cells in low grade, cribriform DCIS. (x50) **2E**- Positive nuclear and/or cytoplasmic immunostain for psoriasin (S100A7). The staining is heterogeneous, with negative and positive areas of DCIS. (x50) 2F- Positive nuclear and/or cytoplasmic immunostain for calgranulin (S100A9). The staining is heterogeneous with some negative and some strongly positive cells within the same focus of DCIS. Note the positive reaction in the granulocytes (arrow, x200).

**Figure 3:**
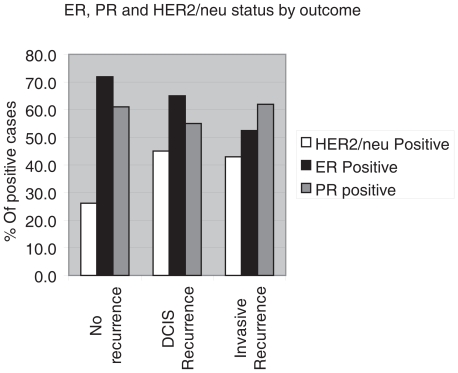
The proportion of Her2/neu overexpressed cases was significantly higher in patients with invasive or in situ recurrence compared with those that never recurred. The difference in ER and PR expression was not significant.

The distribution of percentage of positive cells for the biological markers as demonstrated by immunohistochemistry is shown in Figure 1A-H. When a cut-off for positivity was defined at >10%, the proportion of DCIS showing positive staining for the different tumor markers were as follows: ER (62.4%), PR (55.6%), HER2/neu (31.6%), MIB1 (39.8%), p53 (22.6%), p21 (39.8%), Cyclin D1 (95.5%) calgranulin (20.5%), psoriasin (12%). Representative cases of positive immunostains are shown in Figure 2(A-F). In 20 cases in which HER2/neu immunostain was equivocal, HER2/neu gene amplification was tested by CISH to determine its status, and 4 cases showed HER2/neu gene amplification. HER2/neu oncoprotein overexpression or gene amplification was positive in 45% and 42.9% in cases that recurred as DCIS or invasive cancer respectively, but only in 26.1% of cases that never recurred (p = 0.04). The difference in ER and PR expression between cases that recurred to those that did not recur was not statistically significant (Fig. 3). The correlations between the various immunostains, tumor grade and comedo necrosis are summarized in Table 3. Correlation analysis revealed significant associations between ER-negativity, HER2/neu overexpression, MIB1, p53, p21, calgranulin and psoriasin positivity and high tumor grade (p < 0.01). Comedo necrosis correlated with PR-negativity, HER2/neu overexpression, MIB1 positivity and p53 positivity (p < 0.01). None of the markers correlated with microinvasion. The expression of p21 did not correlate with ER, PR or HER2 status. Calgranulin B and psoriasin immunostains showed marked intratumoral heterogeneity. Most cases showed both positive and negative foci of DCIS (Figs. 2E and 2F). Interestingly, the same foci of DCIS that were positive for psoriasin were also positive for calgranulin, while other foci of DCIS in the same case were negative for both. Calgranulin B and psoriasin expression were highly associated (Pearson coefficient 0.679, p < 0.01); calgranulin B expression was inversely associated with ER and PR expression and directly associated with HER2/neu status. Psoriasin expression showed strong association with high nuclear grade and an inverse association with ER status. Of the 41 patients that recurred, only 4 initial tumors were positive for psoriasin, and all recurrences were non-invasive. In 2 of these cases, HER2/neu was overexpressed. This difference between psoriasin-positive and psoriasin-negative DCIS only approached significance (p = 0.065). On univariate analysis, HER2/neu overexpression was the only marker associated with a higher risk for any recurrence (p = 0.044). The hazard ratio for recurrence for HER2 positive DCIS is 1.927 (confidence interval 1.016–3.653) compared to HER2 negative DCIS. On multivariate analysis HER2/neu overexpression remained the only independent variable significantly associated with any recurrence (p = 0.014) and with invasive recurrence (p = 0.044).

*Comparison between primary DCIS and in situ recurrence* (n = 20; blocks were available in 17 cases): The nuclear grade was concordant in 90% (18/20), and comedo type necrosis in 85% (17/20). The nuclear grade of the 2 discordant recurrent tumors was higher than the primaries. The expression profile of ER/PR HER2/neu was identical in 58.8% (10/17). ER status changed in 2 cases (1 gain and 1 loss). PR status changed in 4 cases (1 gain and 3 losses). Two cases showed loss of HER2/neu overexpression and a third case showed a gain. This third case showed a minor low-grade component featuring similar expression of markers to the primary, and a major high-grade component associated with loss of PR, and gain of HER2/neu overexpression.

*Comparison between primary DCIS and invasive recurrence* (n = 21; blocks were available in 19 cases): The nuclear grade was concordant in 57% (12/21). Of the 9 discordant cases 7 showed a higher nuclear grade in the recurrence. The expression profile of ER/PR HER2/neu was identical in 57.8% (11/19). ER status changed in 4 cases (3 gains and one loss). One case showed loss of PR. In 4 cases there was loss and in one case a gain of HER2/neu overexpression. In one case the loss of HER2/neu overexpression was coupled by gain of PR.

## Discussion

In the current study, we analyzed histological characteristics and a panel of 9 antibodies in a well characterized group of 133 cases of pure DCIS treated in a single cancer center by BCS alone with a long follow up. We performed the immunohistochemical staining on an individual slide rather than using tissue microarray technique to assure optimal representation of small lesions and to evaluate intratumoral heterogeneity. The study has generated a number of observations related to the prognosis of DCIS. None of the morphological parameters including nuclear grade and the presence of comedo necrosis were associated with recurrence in our study. Earlier studies have reported contradicting results with regards to histological features as predictors of recurrence in DCIS. Both the NSABP B-17(6) and EORTC(10) trials, observed a significant association between comedonecrosis and local failure. The role of margin status as an independent risk factor for recurrence continues to be controversial. A recent multivariate analysis(32) of 445 cases of DCIS treated by excision alone showed that margin width was the most significant predictor of local recurrence. The 8 years follow up report on the NSABP B-17 and the 10.5 years follow up report on the NSABP B-24 trials failed to demonstrate that involved margin is a significant predictor of recurrence(20;33). Nuclear grade but not comedo necrosis was also a significant predictor of local recurrence. In an Australian nested case-control study(8), both nuclear grade and extensive necrosis were significant predictors of recurrence. The Australian study included patients managed by BCS or mastectomy with or without adjuvant radiotherapy or hormonal therapy.

In the current study, we have demonstrated for the first time, that HER2/neu is a significant factor that predicts for invasive recurrence independent of tumor grade. In 1988, van de Vijver et al. described overexpression and amplification of *HER-2*/*neu* in carcinoma-in-situ; but did not correlate it with prognosis in his study(34). We found that HER2/neu was positive in 31.6% of the cases. This is concordant with recent studies that demonstrated HER2/neu expression in 32% (n = 95) and 33% (n = 151) of DCIS(26;35;36). Earlier studies reported a higher level of HER2/neu overexpression in 55% of DCIS in general(27;28;37), and in 60%–70% in high grade DCIS(10;38). The higher percentage of positive HER2/neu cases reported earlier could be related to the use of non-standarized methodology or different scoring methodology. We have demonstrated a significant association between HER2/neu over-expression in DCIS and any recurrence and more notably, we have demonstrated a significant association specifically with recurrence as an invasive cancer. HER2/neu overexpression remained a significant predictive factor even when morphologic and other biologic markers investigated in this study were accounted for in multivariate analysis. Our findings are in line with one case-control study(35) that found a significant association between HER2/neu positivity and increased risk for recurrence. However, in that case-control study, the prognostic value of biological markers was examined in DCIS patients that were treated with various modalities and did not analyze their data specifically for invasive type of recurrence. They found that HER2/neu was positive in 41% of DCIS that recurred and in 12% in DCIS that did not recur. The difference in HER2/neu status was significant even when adjusted for high grade (p = 0.01). Other studies showed that HER2/neu positivity correlated with high tumor grade and comedo necrosis, p53 accumulation, and was inversely related to ER, PR and bcl-2 expression, but was not found to be an independent prognostic factor(28;37;39;40). Stark et al. found an association between HER2/neu expression in benign breast lesions and an increased risk of subsequent development of invasive carcinoma. They conducted a case-control study that examined HER2/neu status in biopsies of benign breast disease including non-proliferative and proliferative lesions, atypical ductal hyperplasia and DCIS with a mean follow up of 10.2 years. Gene amplification was detected in 9.5% of the benign specimens and was associated with increased risk of invasive breast cancer. Nevertheless, this association only approached statistical significance. Presently, it is unclear what is the role of HER2/neu overexpression in the pathway of tumor progression. The HER2/neu gene encodes a member of the epidermal growth factor (EGF) receptor family of receptor tyrosine kinases. This receptor has no known ligand binding domain of its own and therefore cannot bind growth factors. However, it does bind tightly to other ligand-bound EGF receptor family members to form homo or heterodimer, stabilizing ligand binding and enhancing kinase-mediated activation of downstream signaling pathways, such as those involving mitogen-activated protein kinase and phosphatidylinositol-3 kinase. It has been suggested that HER2/neu signaling mediates cell motility(41;42). This is supported by studies that examined HER2/neu expression in mammary Paget’s disease(43). It is plausible that the overexpression of HER2/neu in DCIS contributes to cell migration that may play a role in the transition from DCIS to invasive cancer. Based on the observation that the overall incidence of HER2/neu gene amplification/protein overexpression in invasive carcinoma is lower in invasive carcinoma than in DCIS, other investigators suggested that other molecular events might be more critical in the progression from in situ to invasive cancer(36).

S100 Proteins are a group of calcium binding proteins. Their exact biological function is yet to be clarified(44;45). S100A7 protein also known as psoriasin, has been shown to be associated with poor survival in invasive breast cancer(46). We found that psoriasin was expressed only in 12.5% of DCIS. Psoriasin positive cases did not develop an invasive recurrence, even when the tumors were high grade, HER2/neu positive or exhibit comedo necrosis, a feature that has been thought to be associated with increased risk of recurrence. This observation should be addressed cautiously since the number of psoriasin positive cases in our study is low. Previous studies found that psoriasin level was higher in DCIS with invasive carcinoma and demonstrated a high psoriasin expression in DCIS with comedo necrosis(47).

In the present study, calgranulin B (S100A9) another member of the S100 family of proteins, was significantly associated with high nuclear grade, psoriasin positivity and HER2/neu overexpression and was inversely associated with ER and PR. Calgranulin B did not predict for invasive or DCIS recurrence in our study. The association of calgranulin expression and high tumor grade is in line with a previous study that included only 25 cases of DCIS.

Cyclin D1 was expressed almost uniformly in DCIS usually with high proportion of positive tumor cells and did not correlate with prognosis in our study. In earlier studies, Cyclin D1 expression was associated with tumor grade(25), and inversely associated with local recurrence and shorter time to recurrence(48).

Our study showed that 39.8% of DCIS had more than 10% of the cells positive for p21, which is close to the level of expression reported earlier(35). In our study p21 expression did not correlate with patient outcome, or with the other markers. The correlation between p21 and tumor grade was significant at a level of p = 0.05 which was weaker than some of the other markers. There are conflicting reports regarding the association between p21 and both nuclear grade and ER status in DCIS(49). In only one previous study, p21 was shown to be a significant predictor for recurrence independent of the ER/PR/HER2/neu status of the tumor(35). Other studies did not confirm that p21 expression was related to recurrence of DCIS(26;50) and it was not correlated to any other biological marker(26).

In the current study, there was no significant association between ER, PR, MIB1 and P53 expression and recurrence. These results are in accordance with other studies that examined these markers in DCIS treated exclusively by local excision(26;28;40).

When recurrent tumors were compared with their paired primaries, there was a high level of concordance in nuclear grade and comedo necrosis in most tumors that recurred as DCIS; whereas, there was a significant difference in nuclear grade in cases with invasive recurrence (p = 0.018). In both types of recurrences, the discordant cases showed a tendency for recurrent tumors to be of a higher nuclear grade than the primaries. A high level of concordance was maintained when each marker was assessed individually; however, the concordance of the panel of biological markers was noted only in about 60% of the cases. Gain or loss of expression of molecular markers occurred not only in tumors recurring as invasive carcinoma, but also in cases that maintained their in situ histology. These findings are without the impact of radiation or systemic therapy. Our findings suggest that the biological profile should be evaluated on recurrent tumors since they may influence the patient’s treatment(51).

Our study suggests that biological markers as HER2/neu and possibly psoriasin add significant information to the prognosis of DCIS over the currently accepted Van Nuys prognostic index. The results might indicate that HER2/neu testing should be considered in DCIS at the time of diagnosis. Confirmation of these results may initiate a change in the current standard of care for DCIS and specifically to manage patients at an increased risk of recurrence more aggressively. Furthermore, treatment of selected high risk cases with humanized monoclonal antibodies directed against HER2/neu oncoprotein may be considered, and is currently under clinical trial in the neoadjuvant setting(52).

## Figures and Tables

**Table 1:** Antibodies used in the study.

Antibody	Clone	Manufacture	Cat. #	Dilution	Host	Antigen retrieval
ER	6F11	NovaCastra	NCL-L-PGR-312	1:50	Mouse (monoclonal)	HIER Citrate, pH6
PR	312	NovaCastra	NCL-ER-6F11	1:100	Mouse (monoclonal)	HIER Citrate, pH6
HER2	CB11	NovaCastra	NCL-L-CB11	1:400	Mouse (monoclonal)	HIER Citrate, pH6
MIB1	SP6	LabVision	RM-1906	1:400	Rabbit (monoclonal)	HIER Citrate, pH6
Cyclin D1	SP4	Biocare	CP236C	1:100	Rabbit (monoclonal)	HIER Borg, pH9.5
p21	EA10	Oncogene	OP64	1:100	Mouse (monoclonal)	HIER Citrate, pH6
p53	DO7	NovaCastra	NCLP53DO7	1:500	Mouse (monoclonal)	HIER Citrate, pH6
Psoriasin	47C1068	Imgenex	IMG-409A	1:500	Mouse (monoclonal)	HIER Citrate, pH6
Calgranulin B	H-90	Santa Cruz	SC-20173	1:200	Rabbit (polyclonal)	HIER Citrate, pH6

**Table 2:** Clinical and pathological characteristics and recurrence.

	No recurrence N = 92	DCIS recurrence N = 20	Invasive recurrence N = 21
**Age**	57.5	56.6	52
**Mean follow up (years)**	8.3	10.1	10.4
**Nuclear Grade**			
1 n = 18	12 (66.7%)	3 (16.7%)	3 (16.7%)
2 n = 58	44 (75.9%)	5 (8.8%)	9 (15.5%)
3 n = 57	36 (63.2%)	12 (21.1%)	9 (15.8%)
**Comedo Necrosis**			
Absent n = 45	32 (71.7%)	4 (8.9%)	9 (20.0%)
Present n = 87	59 (67.8%)	16 (18.4%)	12 (13.8%)
**Margin**			
<1 mm	37 (40.2%)	8 (40%)	9 (42.8%)
1–10 mm	45 (48.9%)	12 (60%)	10 (47.6%)
>10 mm	7 (7.6%)	0	1 (4.8%)
N/A	3 (3.3%)	0	1 (4.8%)
**Mean Tumor Size (mm)**			
N/A	10.4 ± 5.32	10.6 ± 3.04	8.92 ± 4.56
**ER Positive**	23 (25%)	5 (25%)	6 (28%)
**PR Positive**	61 (66%)	13 (65%)	12 (57%)
**HER2/neu**	53 (58%)	11(55%)	13 (62%)
**Overexpressed**	24 (26%)	9 (45%)	9 (43%)

**Table 3:** Associations between the biological markers and histopathological features, Pearson correlation coefficient

	High Grade	Comedo Necrosis	Percentage of positive tumor cells							
			ER	PR	HER2 Positive	Cyclin D1	p21	MIB1	Calgranulin	Psoriasin
Comedo Necrosis	0.401**									
ER % Cells +ve	−0.337**	−0.198*								
PR % Cells +ve	−0.15	−0.254**	0.485**							
HER2 Positive	0.294**	0.354**	−0.452**	−0.325**						
Cyclin D1 % Cells +ve	−0.124	0.016	0.356**	0.250**	−0.125					
p21 % Cells +ve	0.218*	0.181*	−0.146	−0.290**	0.136	0.264**				
MIB1 % Cells +ve	0.432**	0.425**	−0.111	−0.213*	0.228**	−0.037	0.119			
Calgranulin % Cells +ve	0.304**	0.143	−0.428**	−0.355**	0.311**	−0.315**	0.037	0.145		
Psoriasin % Cells +ve	0.257**	0.163	−0.251**	−0.179*	0.121	−0.319**	−0.039	0.109	0.679**	
P53 % Cells +ve	0.252**	0.264**	−0.238**	−0.238**	0.316**	−0.174*	−0.188*	0.337**	0.228**	0.054

** Correlation is significant at the 0.01 level (2-tailed).

* Correlation is significant at the 0.05 level (2-tailed).
